# Can robotic gastric bypass be considered a valid alternative to laparoscopy? Our early experience and literature review

**DOI:** 10.3389/fsurg.2024.1303351

**Published:** 2024-02-05

**Authors:** Giovanna Pavone, Mario Pacilli, Alberto Gerundo, Andrea Quazzico, Antonio Ambrosi, Nicola Tartaglia

**Affiliations:** Department of Medical and Surgical Sciences, University of Foggia, Foggia, Italy

**Keywords:** Roux-en-Y gastric bypass, bariatric surgery, robotic bariatric surgery, laparoscopic surgery, da Vinci robot®

## Abstract

**Background:**

Robotic bariatric surgery serves as an alternative to laparoscopy. The technology provides the surgeon with an accurate three-dimensional view, allowing complex maneuvers while maintaining full control of the operating room.

**Hypothesis:**

We report our experience with this innovative surgery compared with laparoscopy during Roux-en-Y gastric bypass to demonstrate its safety and feasibility. The aim of this study is to evaluate potential differences between the robotic and laparoscopic techniques.

**Materials and methods:**

Our study retrospectively identified 153 consecutive obese patients who underwent either laparoscopic or robotic gastric bypass (RGB) procedures over a 2-year period at the Department of Medical and Surgical Sciences, University of Foggia. Data on demographics, operative time, conversion rate, length of hospital stay, and mortality were collected and compared between two groups of patients: 82 patients who underwent laparoscopic procedures and 71 who underwent robotic procedures.

**Results:**

We analyzed 153 patients who underwent gastric bypass with a mean age of 42.58 years, of whom 74 were female; 71 were treated with a robotic approach and 82 with a laparoscopic approach. The mean operative time was 224.75 ± 10.4 min for RGB (including docking time) and 101.22 min for laparoscopic gastric bypass (LGB) (*p* < 0.05), which is statistically significant. The median length of stay was 4.1 days for the RGB group and 3.9 days for the LGB group (*p* = 0.89). There is only one conversion to laparoscopy in the RGB group. We observed only one case of postoperative complications, specifically one episode of endoluminal bleeding in the laparoscopic group, which was successfully managed with medical treatment. No mortality was observed in either group.

**Conclusion:**

The statistical analysis shows to support the robotic approach that had a lower incidence of complications but a longer operative duration. Based on our experience, the laparoscopic approach remains a technique with more haptic feedback than the robotic approach, making surgeons feel more confident.

This study has been registered on ClinicalTrial.gov Protocol Registration and Results System with this ID: NCT05746936 for the Organization UFoggia (https://clinicaltrials.gov/ct2/show/NCT05746936).

## Introduction

Obesity is a chronic, relapsing disease associated with numerous complications and significant morbidity, mortality, and healthcare burden. Bariatric surgery now plays an important role in the treatment of obesity in addition to non-invasive conservative treatments such as lifestyle changes, pharmacotherapy, and behavioral therapy (1, 2). In 1991, the National Institutes of Health Consensus Statement on Gastrointestinal Surgery for Severe Obesity stated that bariatric surgery is the most effective treatment for obesity owing to its benefits in weight loss, glycemic control, and reduced mortality (3, 4).

Roux-en-Y gastric bypass (RYGB) is the most common procedure in Europe to treat severely obese people (5, 6), particularly in the presence of gastroesophageal reflux or type 2 diabetes cases (7–9).

The laparoscopic technique is the most widely used for Roux-en-Y gastric bypass, which is significantly superior to open surgery (10, 11), with a low complication rate but high technical requirements and a flat learning curve between 1,006 and 500 cases (12, 13).

However, the da Vinci® Surgical System (Intuitive Surgical Inc., Sunnyvale, CA, USA), which was introduced in 2000, successfully addressed certain technical limitations associated with laparoscopic surgery. One of the main advantages is the improved dexterity of the instrument (increased freedom of movement) and the filtering of tremors, which allow the surgeon to operate as if in open surgery, allowing delicate microsurgery and microanastomosis (14, 15). Other advantages include a three-dimensional view of the surgical field that provides better depth perception, the ability for the surgeon to control the field of view of the surgical field, and an ergonomically designed workstation that allows the surgeon a comfortable sitting position (16, 17).

In this study, we report our experience with this innovative surgery compared with laparoscopy during Roux-en-Y gastric bypass to demonstrate its safety and feasibility. The aim is to evaluate if any differences exist between the robotic and laparoscopic techniques.

## Materials and methods

Our study retrospectively identified 153 consecutive obese patients who underwent either laparoscopic or robotic gastric bypass (RGB) procedures over a 2-year period (2020–2022) at the Department of Medical and Surgical Sciences, University of Foggia. Data on demographics, operative time, conversion rate, length of hospital stay, and mortality were collected and compared between two groups of patients: 82 patients who underwent laparoscopic procedures and 71 patients who underwent robotic procedures.

The unique protocol identification number (UIN) for the ClinicalTrial.gov Protocol Registration and Results System is NCT05746936 for the Organization UFoggia (https://clinicaltrials.gov/ct2/show/NCT05746936).

### Inclusion criteria

Individuals who have a body mass index (BMI) of ≥ 35–39 kg/m^2^ and at least one obesity-associated comorbidity or a BMI of ≥ 40 kg/m^2^ and are 18 years of age or older were included in the study. Prior to surgery, the patients underwent a standardized psychological and physical evaluation that included blood chemistry tests, chest x-rays, electrocardiogram and cardiological examinations, nutritional evaluation, esophagogastroduodenoscopy, spirometry, and psychiatric evaluation.

### Exclusion criteria

We excluded re-do surgery procedures from the study.

### Statistical analysis

Continuous data were expressed as mean and standard deviation (SD), and statistical analysis was performed using the *χ*^2^ test with a *p*-value threshold of less than 0.05 (*p* < 0.05) and the Mann–Whitney *U* test to determine statistical significance.

### Surgical technique

**Figure 1:**
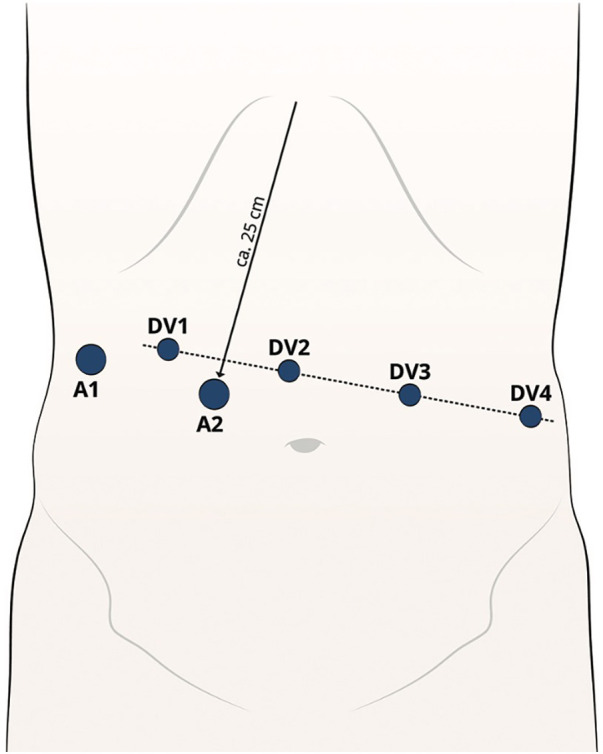
Robotic trocar placement.

A pneumoperitoneum is established (15 mmHg) using a 12 mm First Entry trocar, positioned approximately 25 cm below the xiphoid [Assistant trocar 2 (A2)] on the right paramedian side. Another 12 or 5 mm (depending on the retractor) trocar [Assistant trocar 1 (A1)] is inserted on the right lateral flank. This is mainly used for liver retractions. From A1, a line is drawn across the upper abdomen at a 90° angle to the xiphoid-A2 line. All da Vinci (DV) ports are positioned along this line. Port 1 (8 mm) (DV1) is positioned 8–10 cm lateral to port A1. Port 2 (8 mm) (DV2) is positioned 8–10 cm to the side of port 1. This will be the camera port. Port 3 (8 mm) (DV3) and port 4 (8 mm) are positioned (DV4) 8–10 cm lateral to each other on the patient's left side (Figure 1) (18).

The liver paddle is first inserted through the A1, and the liver is retracted toward the right upper quadrant.

Starting from the ligament of Treitz, 120 cm of jejunum is measured aborally, and the jejunum is dissected by the assistant through a linear stapler from A2, creating the biliary limb. Another 120 cm is counted (alimentary limb), and the jejunum is opened antimesenterally on both limbs with Ultracision, and a side-to-side jejunojejunal anastomosis is created using the 60 mm linear stapler. The enterotomy is closed using a single suture with Stratafix 3-0 in a seromuscular suture.

To preserve the left gastric artery, a retrogastric tunnel is formed starting 6 cm below the lesser curvature gastroesophageal junction. In order to minimize the risk of stricture or dumping syndrome, the anesthesiologist inserts a 40 mm bougie orally to assure the appropriate size of the pouch. Using a linear stapler, the stomach pouch is formed with the bougie as a calibration.

Ultracision is used to open the pouch at its lowest point, away from the minor curvature.

The stapler is inserted into the gastric pouch and the alimentary limb, and then the lateral gastrojejunal anastomosis is contoured, ensuring good positioning of both ends to create only a small enterostomy.

A 15 cm unidirectional Stratafix 2-0 suture is used to close the enterotomy for a continuous seromuscular suture. Arm 4 is used to optimize the position of the small bowel loop, while arms 1 and 3 are used dynamically for suturing. Starting on each side of the enterostomy, two sutures are required to complete the anastomosis.

A methylene blue test is conducted to test the gastrojejunostomy. The systolic blood pressure is then raised above 130 mmHg to identify any signs of bleeding. Any bleeding is treated using either the bipolar forceps, with a clip application, or a suture.

A drainage is placed through the A1, and all trocars are retrieved under view. The pneumoperitoneum is released although the A2 and the patient cart can be undocked.

## Results

We analyzed 153 patients who underwent gastric bypass, 74 of whom were female, with a mean age of 42.58 years. Among the patients, 71 were treated using a robotic approach, while 82 were treated using a laparoscopic approach. The initial mean weight was 130.50 kg, and the initial mean body mass index was 45.73 kg/m^2^, specifically 43.68 ± 6.1 kg/m^2^ for the robotic group and 45.53 ± 7.1 kg/m^2^ for the laparoscopic group.

The mean operative time was 224.75 ± 10.4 min for robotic gastric bypass (including docking time) and 101.22 min for laparoscopic gastric bypass (LGB) (*p* < 0.05), which is statistically significant (Table 1). The z-score was −5.92815, and the value of *U* was 0, indicating that the distribution is approximately normal.

**Table 1:** Preoperative and intraoperative characteristics and comorbidities of the study groups.

	Robotic Group	Laparoscopic Group	*χ*^2^ test –*p*-value <0.05	Mann–Whitney *U* test
Age (years) mean ± (SD)	46.58 ± 6.4	39.5 ± 8.2	n.s	n.s
Female number (%)	35 (49.3%)	39 (47.6%)	n.s	n.s
Preoperative mean BMI (kg/m^2^) ± (SD)	43.68 ± 6.1	45.53 ± 7.1	n.s	n.s
Operative time (minutes) ± (SD)	224.75 ± 10.4	101.22 ± 8.2	*p* < 0.05	−5.92815
Median length of stay (days) ± (SD)	4.1 ± 3.1	3.9 ± 2.6	*p* = 0.089	0.18301
Rate of conversions number (%)	1 (1.4%)	0 (0%)	//	//
Complication number (%)	0 (0%)	1 (1.2%)	//	//
Operating room time costs (€)	18,421.07	2,088.09	*p* < 0.05	2.17218

SD, standard deviation; n.s., not significant.

**Figure 2:**
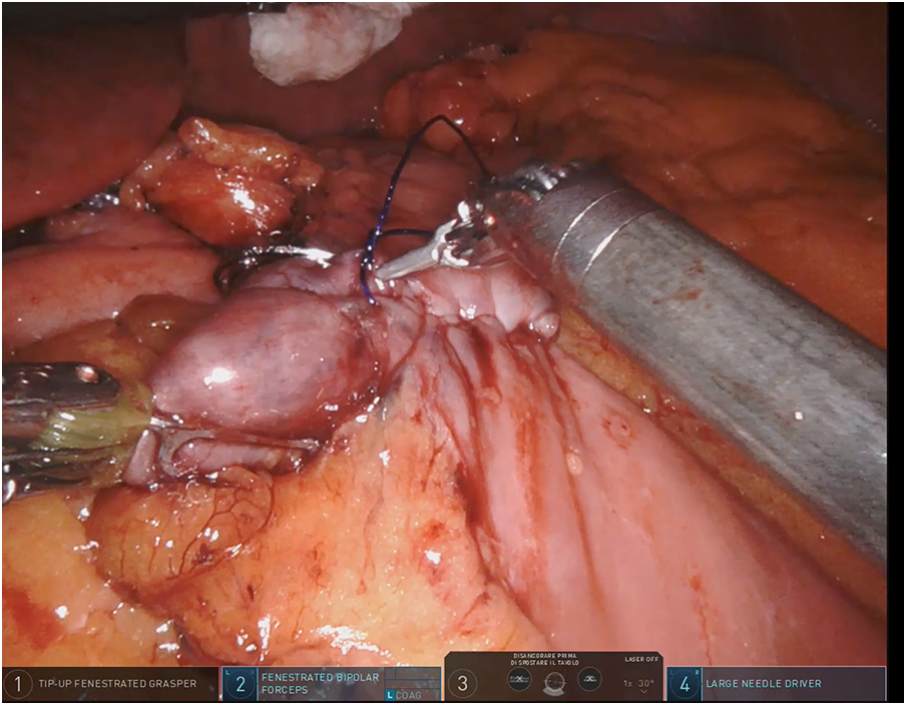
Robotic gastrojejunal anastomosis.

**Figure 3:**
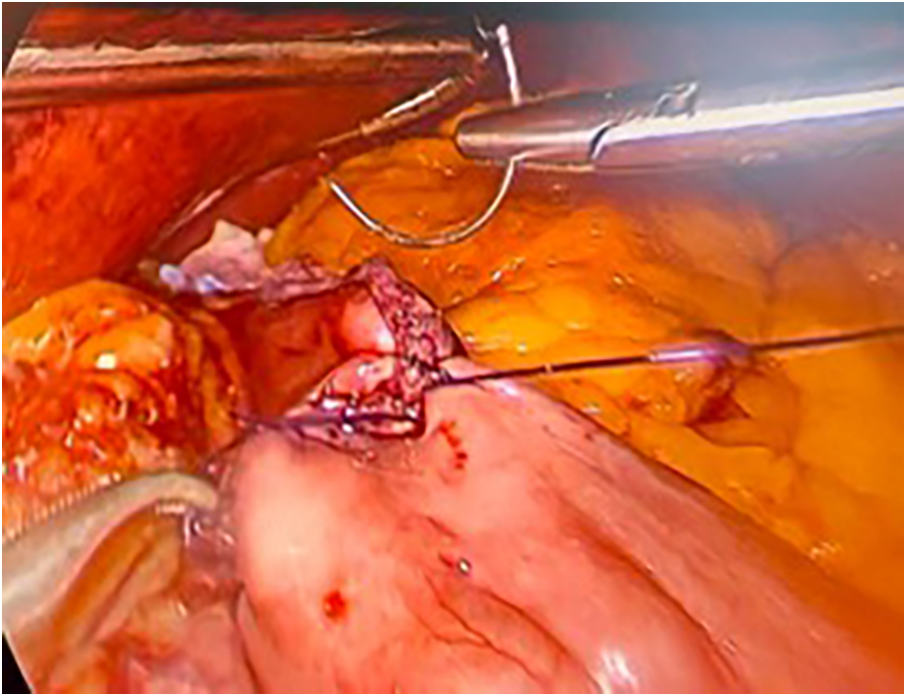
Laparoscopic gastrojejunal anastomosis.

The surgical technique is the same, which provides for the creation of a gastric pouch, biliopancreatic limb, jejunojejunal anastomosis, alimentary limb, and gastrojejunal anastomosis (Figures 2,3).

The median length of hospital stay was 4.1 days for the RGB group and 3.9 days for the LGB group (*p* = 0.89). The z-score was 0.18301, and the value of *U* was 201.5, indicating that the distribution is approximately normal.

Patients were discharged on the third postoperative day after oral contrast gastrointestinal tract radiography.

**Figure 4:**
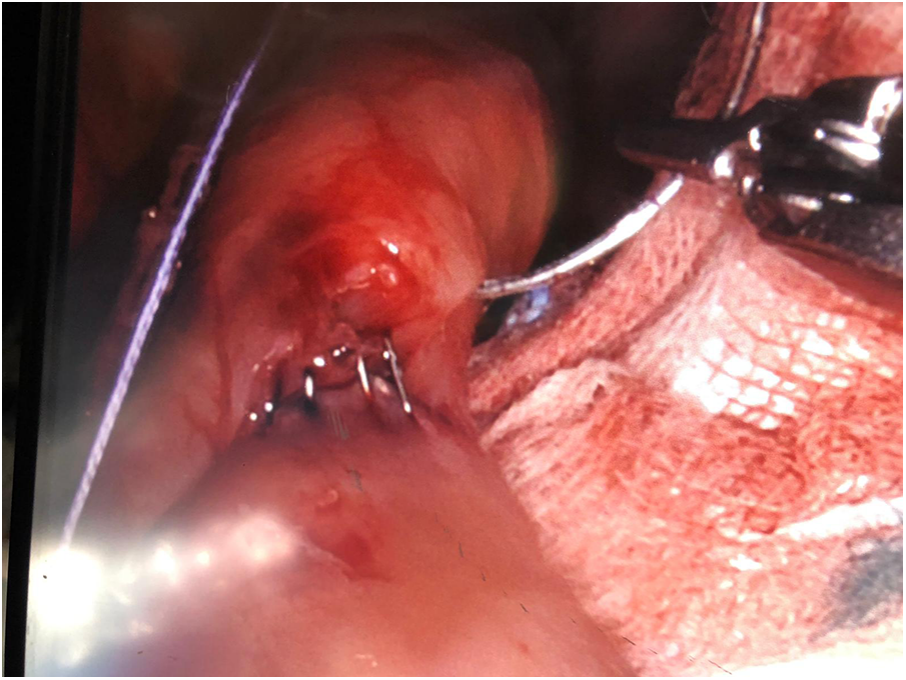
Positive intraoperative methylene blue test on the gastrojejunal anastomosis.

**Figure 5:**
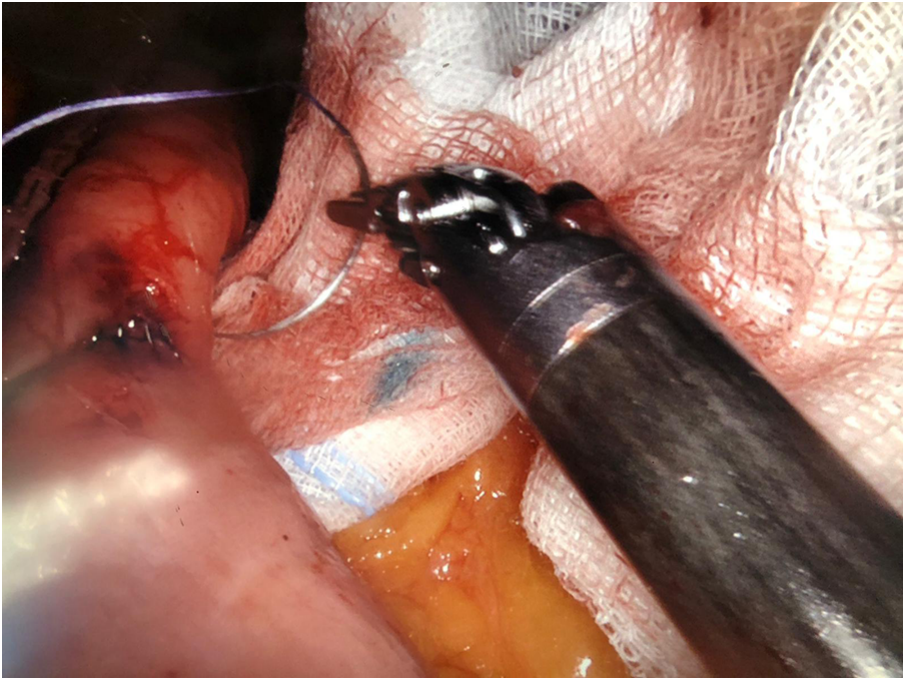
Gastrojejunal anastomosis leak.

There was only one case of converting to laparoscopy in the robotic group due to the detection of a positive intraoperative methylene blue test. The surgeon preferred to reinforce the gastrojejunal anastomosis with laparoscopic sutures due to the lack of haptic feedback with the robotic procedure. We observed only one case of postoperative complications: one occurrence of endoluminal bleeding in the laparoscopic group, which was successfully managed with medical treatment. There were no deaths observed in either group (Figures 4,5).

The operative time of the RGB group was longer than that of the LGB group, resulting in a significantly higher operating room time costs (median €18421.07 vs. €2088.09, respectively, *p* < 0.005).

## Discussion

For approximately 20 years, robotic surgery has been successfully used in critical bowel, gastric, pancreatic, urological, and transplant procedures, where anastomoses are a critical part of the procedure as they are often time-sensitive, particularly when warm ischemia must be minimized, such as during transplant surgery. Organs are critical to transplant function and outcome (19–21). In the literature, robotic approaches have provided similar results to laparoscopic and open approaches in terms of anastomotic leaks or strictures. There was no significant difference found in leakage after RYGB when comparing robotic anastomosis with laparoscopic anastomosis. Robotic surgery is gaining popularity and offers solutions to the challenges of laparoscopy, including ergonomics, high-resolution 3D cameras, tremor filtering, the surgeon's third arm, and handheld instruments (22).

In the bariatric surgical field, for example, these features translate into the ability to perform better traction of a normally thick abdominal wall, relieving the surgeon's physical efforts to overcome the counterproductive forces, as well as a highly stable camera and better manipulation of the surgical structures (23).

In agreement with the literature data, our study did not show significant differences in terms of the length of hospital stay, reoperations, and mortality (24, 25).

The mean operative time was significantly shortened (101.22 min) in the laparoscopic group due to the time-consuming setup and docking phase featuring the robotic approach.

There was only one case of conversion from a robotic to a laparoscopic approach, probably due to the greater haptic feedback guaranteed by laparoscopy.

Compared with endoscopic or laparoscopic techniques, robotic surgical systems suffer from a complete loss of tactile feedback to the user (26). Consequently, surgeons rely on visual cues and their expertise in order to perform the accurate motor movements required for operations (27, 28). The absence of tactile feedback leads to prolonged procedural times and a greater risk of surgical error (29).

There was only one complication in the laparoscopic group, which can be explained by the better accuracy and precision of the intracorporeal suture during the robotic approach in comparison with the traditional laparoscopic approach.

Our results are consistent with the data presented by Wilson et al. (30) at the 2012 American Society for Metabolic and Bariatric Surgery Annual Meeting in San Diego. The study involved the enrollment of 1,695 patients who underwent robotic RYGB surgery. The postoperative complications included ileus in 17 patients, wound infection in five patients, and bleeding in 18 patients. The readmission rate was 4.8%, and the reoperation rate was 2.7%. The rates of leakage and anastomotic stenosis were extremely low, at 0.3% and 0.2%, respectively. There were no deaths reported. “This largest series of robotic bypass reports from three high-volume centers shows very low complication rates in the first 30 days. It shows zero 30-day mortality, very low leak rates, and provides a strong evidence that Robotic GB can deliver extremely safe and reproducible results.”

Kim et al. (31, 32) concluded that the use of the robot is associated with a shorter learning curve, particularly in performing delicate and precise maneuvers such as fine dissections and sutures. Indeed, it is widely recognized that robotic bariatric surgery, particularly RGB, has a steeper learning curve than the laparoscopic approach, and a minimum of 20 cases may be sufficient to successfully complete the basic learning phase.

The most important limitation of this study is the small sample size of patients, but despite this, it offers a preliminary statistical analysis between the two procedures. Furthermore, the learning curve can contribute to a decrease in the duration of a robotic gastric bypass procedure.

## Conclusion

Our statistical analysis seems to favor the robotic approach due to a lower incidence of complications, but the major disadvantage of the robotic bariatric surgery still remains the long operative time.

In our experience, the laparoscopic approach continues to be a technique that provides greater haptic feedback compared with the robotic approach, making the surgeon feel more confident. RGB was found to be comparable with LGB in terms of safety and efficacy, but larger and longer studies are needed for a better comparative evaluation.

## Data Availability

The original contributions presented in the study are included in the article/Supplementary Materials; further inquiries can be directed to the corresponding author.
